# The prevalence of HEV among non-A-C hepatitis in Qatar and efficiency of serological markers for the diagnosis of hepatitis E

**DOI:** 10.1186/s12876-021-01841-2

**Published:** 2021-06-15

**Authors:** Enas S. Al Absi, Duaa W. Al-Sadeq, Makiyeh Khalili, Nadin Younes, Nader Al-Dewik, Sara K. Abdelghany, Somaia S. Abouzid, Asma A. Al Thani, Hadi M. Yassine, Peter V. Coyle, Gheyath K. Nasrallah

**Affiliations:** 1 Biomedical Research Center, Qatar University, P.O. Box 2713, Doha, Qatar; 2 College of Medicine, Member of QU Health, Qatar University, P.O. Box 2713, Doha, Qatar; 3 Department of Laboratory Medicine, Hamad Medical Corporation, Doha, Qatar; 4 Clinical and Metabolic Genetics Section, Pediatrics Department, Hamad General Hospital, Hamad Medical Corporation, P.O. Box 3050, Doha, Qatar; 5 Qatar Medical Genetic Center and Interim Translational Research Institute, Hamad Medical Corporation, P.O. Box 3050, Doha, Qatar; 6 College of Health and Life Science, Hamad Bin Khalifa University, P.O. Box 34110, Doha, Qatar; 7 Department of Pediatrics, Women’s Wellness and Research Center, Hamad Medical Corporation, P.O. Box 3050, Doha, Qatar; 8 Department of Biomedical Science, College of Health Sciences, Member of QU Health, Qatar University, Women’s Science Building, C01, P.O. Box 2713, Doha, Qatar; 9 Virology Laboratory, Hamad Medical Corporation, P.O. Box 3050, Doha, Qatar

**Keywords:** Non-A-C hepatitis, RT-PCR, Wantai ELISA, Test performance, Prevalence, Qatar

## Abstract

**Background:**

The rapid growth of Qatar in the last two decades has attracted a large influx of immigrant workers who mostly come from HEV-hyperendemic countries. Thus, we aim to investigate the prevalence of HEV among acute non-A-C hepatitis patients in Qatar; and to evaluate the performance of four dominant commercial serological assays for HEV diagnosis.

**Methods:**

259 patients with non-A-C hepatitis were tested using the Wantai HEV-IgM, HEV-IgG, HEV-Ag ELISA kits, and the MP Biomedical HEV-Total Ab ELISA kit. ALT levels were tested and HEV RNA (viral loads) was performed using Taqman AmpliCube HEV RT-PCR kit (Mikrogen, Neuried, Germany). The performance of each kit was assessed according to the RT-PCR results.

**Results:**

HEV-RNA was detected in 23.1% of the samples. Most of these HEV-RNA-positive cases belonged to non-Qatari residents from the Indian subcontinent; India, Pakistan, etc. HEV-Ag, HEV-IgM, HEV-IgG, HEV-Total Ab were detected in 5.56%, 8.65%, 32.1%, and 34.2% of all tested samples, respectively. Elevated ALT levels were highly correlated with the HEV-Ag, HEV-IgM, HEV-RNA but not with the HEV-IgG and HEV-Total Ab. Although HEV-Ag was very specific (100%), yet its sensitivity was poor (36.7%). HEV-IgM demonstrated the best second marker for diagnosis of acute HEV after RT-PCR as jugged by the overall performance parameters: specificity (96.2%), sensitivity (71.4%), PPV (83.3%), NPP (92.7%), agreement with RT-PCR (91.0%), and Kappa-value (0.71).

**Conclusion:**

Our study demonstrated a high prevalence of HEV virus in Qatar, mostly among immigrants from the Indian subcontinent. The HEV-IgM represents the best marker for detecting the acute HEV infection, where RT-PCR cannot be performed.

**Supplementary Information:**

The online version contains supplementary material available at 10.1186/s12876-021-01841-2.

## Background

HEV was first identified in Afghanistan in 1983 [1]. It is a single-stranded RNA, non-enveloped, and the only member of the genus Herpesvirus in the Hepeviridae family [2]. Hepatitis E is one of the leading causes of acute liver inflammation globally [3, 4]. According to the World Health Organization (WHO), there are an estimated 20 million HEV infections worldwide every year, leading to an estimated 3.3 million symptomatic cases of hepatitis E globally [5]. In addition, WHO estimates that hepatitis E caused approximately 44,000 deaths in 2015 (accounting for 3.3% of the mortality rate due to viral hepatitis) [5]. Furthermore, HEV constitutes a major concern for public health, especially in developing countries [6–8]. Even though Hepatitis E is a self-limiting disease, it may develop into acute fulminant hepatitis (acute liver failure) [5]. Unlike other forms of viral hepatitis, HEV infection in pregnant women induces a high rate of mortality ranging from 15 to 20% [3, 7, 9]. This virus is predominantly transmitted through the fecal–oral route. However, other routes have been recently identified, including vertical transmission and blood transfusion [10, 11].

HEV is a major public health problem in the Middle East, where its prevalence ranged from 2.0 to 37.5% and higher in males than in females [8]. Studies in Saudi Arabia showed a prevalence of HEV surface antigen ranging from 7.4 to 17%, denoting high endemicity [12, 13]. In addition, a study performed in Dubai-UAE in 2006 and 2007 revealed that 40% of the acute hepatitis cases had HEV infection [14]. Moreover, in the Dakhliya region in Oman, screening of all cases of acute hepatitis in 2003 and 2004 revealed that 12 of 73 (16.4%) confirmed viral hepatitis cases were positive for HEV [15]. However, data on the seroprevalence of HEV antibodies in Qatar is scarce due to the lack of enough epidemiological data.

With the anticipation of the FIFA World Cup 2022, Qatar has seen a spurt of foreign labor with expatriates constituting 95% of a total labor force from over 90 countries [16–21]. The majority of these workers usually come from highly HEV-endemic regions like India, Nepal, Bangladesh, Philippine, Pakistan Sudan, and Egypt. We had shown that the prevalence of anti-HEV IgG and IgM among blood donors of some of these nationalities is more three folds of the local people [22]. Besides, poor sanitation and hygiene as well as sharing accommodation among this group of population increase the risk of transmitting acute hepatitis infection. Recently, more evidence has been accumulating for the direct person-to-person transmission of HEV and nearly 80% of cases could be from households with more than one case [23, 24]. This suggests that this group of immigrants increase the chances of spreading communicable diseases in the community, such as HEV. Thus, we strongly believe that most of the HEV cases in Qatar were also imported from outside through a high influx of migrant workers to Qatar or by traveling back and forth to these highly HEV-endemic areas. Therefore, in this study, we aimed to investigate the prevalence of HEV among non-A-C hepatitis in Qatar.

HEV-RNA detection by RT-PCR remains the gold standard to uncover the true HEV viremia and acute infection, particularly in asymptomatic cases [25, 26]. However, there is a pressing need for identifying reliable immunoassays with high sensitivity and specificity for serology testing and surveillance of HEV infection to be used as a complementary test to RT-PCR to improve its diagnostic sensitivity. Recently, an enzyme immune-assay (ELISA) for HEV-Ag detection was released into the market. The sensitivity results for this assay was very controversial. Some reported low [11] and others reported high [27, 28] concordance with the HEV-RNA detection RT-PCR assay. The clinical impact of hepatitis E in Qatar remains to be clarified, as the increased sensitivity and specificity of the last generation assays suggest a reassessment of previous percentages. Therefore, in the present study, we evaluated the performance of four dominant, commercially available serological assay kits for detecting anti-HEV-IgG, HEV-IgM, HEV-Ag, HEV-Total Ab in samples from 259 non-A-C hepatitis patients (Additional file 1: Table S1). We also compared the sensitivity, specificity, predictive values, and Cohen's Kappa of these kits in relation to RT-PCR. A strength of this study is that it was conducted on a very diverse population reflecting the diversity of the population of Qatar.

## Methods

### Ethical approval

This study was conducted in full accordance with the regulation of research at Hamad Medical Corporation (HMC) and Qatar University (QU). HMC-Institutional Review Board (HMC-IRB #14292/14) and QU-IRB (#556-EA/16) were obtained before sample collection.

### Sample collection and description

From March 2017 to September 2019, 259 anonymous leftover serum samples were collected from the clinical virology lab at HMC. These samples belonged to clinically suspected hepatitis patients. They were classified by the clinical virology lab as suspected non-A-C viral hepatitis patients, because of the negative serology for anti-HAV-IgM, anti-HBc IgM, and HCV-RNA. Hepatitis caused by other diseases, such as autoimmune and drug-induced hepatitis, were not excluded from the study. Patients were not contacted or recruited. Only basic demographic data such as age, gender, and nationality were only collected from these patients.

### Detection of HEV-RNA using Real-Time PCR

RNA was extracted from 200 μL aliquots of serum using a standard qualitative commercial kit for viral RNA extraction from body fluids (QIAamp® Viral RNA Mini Kit Qiagen; Hilden, Germany) according to the manufacturer’s instructions. Reverse transcription and amplification of HEV RNA were performed from 10 μL of the extracted RNA according to the manufacturer’s instructions of the AmpliCube HEV RT-PCR kit (Mikrogen, Neuried, Germany) using QuantStudio™ 6 Flex Real-Time PCR instrument (Applied Biosystems, USA). The cycle threshold (CT) was calculated according to the manufacture’s instruction. Samples with CT value more than 40 were considered negative.

### Detection of HEV-Ag, HEV-IgG, HEV-IgM, and HEV-Total Ab by ELISA

All serum samples were screened for the presence of the HEV-Ag using the Wantai HEV-Ag ELISA kit (Cat. no. WE-7596, Wantai Biological Pharmacy Enterprise Co., Ltd., Beijing, China), HEV-IgM using Wantai HEV-IgM kit (Cat. no. WE-7196, Wantai Biological Pharmacy Enterprise Co., Ltd., Beijing, China) and HEV-IgG using Wantai HEV-IgG kit (Cat. no. WE-7296, Wantai Biological Pharmacy Enterprise Co., Ltd., Beijing, China) according to the manufactures’ instructions. Besides, MP Diagnostics ELISA kit was used to detect HEV total antibodies, which are HEV-IgG, HEV-IgA, and HEV-IgM. The cut-off value of the tests were defined by the positive and the negative control sera that were included in each kit.

### Measuring alanine aminotransferase (ALT)

ALT level is one of many factors that give insight as to whether HEV infections are acute or chronic. The ALT was measured using Greiner Diagnostic ALAT / GP (GmbH - Unter Gereuth 10 – D-79353 Bahlingen – Germany). A total of 20 µLof each sample were used as per manufacturer’s instructions. Samples were incubated with 200 µL of stock solution previously prepared from reagent 1 and reagent 2 for three minutes. Regent 1 contained Tris-buffer (pH 7.5), l-alanine and lactate dehydrogenase (LDH), while reagent 2 contained 2-oxogutarate and NADH. The absorbance was read at 334 nm after each minute of incubation. Subsequently, the delta of the three absorbance results was obtained, and the final results were obtained as follows: $\Delta _{1} = R_{3} - R_{2}$; $\Delta _{2} = R_{2} - R_{1}$; $Activity~\left( {\frac{U}{L}} \right) = \left( {\Delta 1 + \Delta 2~} \right)*1780$. Results were interpreted according to the reference values provided by the manufacturer. Female subjects with ALT levels < 31 U/L were considered normal while male subjects with ALT values < 41 U/L were considered normal.

### Statistical analysis

The diagnostic assessment of the four assays with RT-PCR for HEV resulted in four cross-tabulations. Using RT-PCR as the reference standard, overall percent agreement, sensitivity, specificity, positive predictive value (PPV), negative predictive value (NPV), and Cohen’s kappa statistic were calculated to assess the performance of each assay. The overall agreement between two assays usually measured by the overall percent agreement and Cohen’s kappa. The overall percent agreement is the percentage of total subjects where the new test and the non-reference standard agree. Whereas, Cohen’s kappa statistic is a standard and robust metric that estimates the level of agreement (beyond chance) between two diagnostic tests. Ranging between 0 and 1, a kappa value below 0 denotes no agreement, a kappa value between 0.00 and 0.20 denotes slight agreement, a kappa value 0.21 and 0.40 fair agreement, a kappa value between 0.41 and 0.60 denotes moderate agreement, a kappa value between 0.61 and 0.80 denotes substantial agreement, and a value between 0.81 and 1.00 denotes an almost perfect agreement [29]. We considered all borderline samples to be positive as informed by literature [30, 31]. The significance level was indicated at 5%, and a 95% confidence interval (CI) was reported for each metric. All calculations were performed using Microsoft Excel 2016 and GraphPad Prism 8 software (version 8.2.1).

## Results

### Patients characteristics

A total of 259 serum samples of patients with suspected non-A-C hepatitis were anonymously collected from the clinical virology lab at HMC. The mean age in our studies patients was 39.4 ± 14.92 with the youngest age being 6 and the oldest being 98 years old. Approximately 50% of the patients were aged 25–44 years. 61.4% of the samples belonged to males, while the rest were from females. The relative distribution of samples by nationality showed that Indians (20.8%) comprised the largest percentage, followed by Qataris (20.5%), Nepalese (9.65%) and Egyptians (9.27%) and the rest were from other nationalities. Table 1 highlights the main demographic characteristics of non-A-C patients’ samples.

**Table 1:** Characteristics of the study samples

	All participants (%)	HEV-IgG (%)	*p*-value	HEV-IgM (%)	*p*-value	HEV-Ag (%)	*p*-value	HEV-Total (%)	*p*-value	RT-PCR (%)	*p*-value
Sex											
Male	159 (61.4%)	54 (38.3%)	***p* = 0.01**	16 (11.1%)	***p* = 0.00001**	9 (8.10%)	*p* > 0.05	37 (41.6%)	***p* = 0.025**	20 (28.6%)	***p* = 0.02**
Female	100 (38.6%)	20 (22.4%)		4 (4.5%)		1 (1.45%)		16 (24.2%)		4 (11.8%)	
Age											
0–24	38 (14.7%)	11(28.9%)	***p* = 0.024**	8 (21.1%)	***p* = 0.026**	5 (13.1%)	***p* = 0.024**	9 (23.7%)	***p* = 0.000011**	7 (19.4%)	*p* > 0.05
25–34	65 (25.1%)	14 (21.9%)		3 (4.68%)		2 (30.8%)		9 (14.1%)		7 (25%)	
35–44	65 (25.1%)	15 (23.0%)		5 (7.69%)		1 (1.53%)		9 (20.45%)		4 (14.3%)	
45–54	40 (15.4%)	12 (30.0%)		4 (10.0%)		2 (8.33%)		11 (52.4%)		4 (40%)	
55+	40 (15.4%)	20 (50%)		0		0		15 (62.5%)		2 (22.2%)	
NR	11 (4.2%)	2 (18.1%)		0		0		0		0	
Nationality											
Indian subcontinent	121 (46.7%)	42 (38.8%)	***p* = 0.03**	16 (14.6%)	***p* = 0.02**	9 (10.3%)	***p*** > 0.05	31 (43.7%)	***p* = 0.02**	19 (36.5%)	***p* = 0.01**
West Asia	79 (30.5%)	16 (23.2%)		1 (1.42%)		1 (1.96%)		10 (22.2%)		2 (6.89%)	
South East Asia	12 (4.63%)	3 (27.3%)		1 (10%)		0		0		0	
North Africa	36 (13.9%)	13 (39.4%)		2 (6.25%)		0		11 (40.7%)		3 (23.1%)	
Others	11 (4.25%)	0		0		0		1 (16.7%)		0	
Total positive (%)	74 (32.1)			20 (8.65)		10 (5.56)		53 (34.2)		24 (23.1)	

Bold *p*-value represents value < 0.05, which means significant

### HEV-RNA, HEV-Ag, HEV-IgG, HEV-IgM, and HEV-Total Ab, and ALT results

RT-PCR testing is considered the gold standard test for the diagnosis of acute HEV in immunocompromised patients [32, 33]. Whereas, serology in combination with RT-PCR are recommended for HEV diagnosis in immunocompetent patients [32]. The HEV-RNA was identified by RT-PCR in 23.1% of the samples. The HEV-Ag, HEV-IgG, and HEV-IgM markers were identified by Wantai ELISA kits in 5.56%, 32.1%, and 8.65% of the samples, respectively. While HEV-Total Ab were identified by MP Diagnostics ELISA in 34.2% samples.

ALT values were available for 235 out of 259 specimens. Interestingly, the median ALT values for the HEV-RNA positive specimens was 379 IU/L (95–1314 IU/L), which is almost three-fold greater than negative HEV-RNA specimens (86.5 IU/L (23.5–277 IU/L)). Only one of the positive RNA specimens (No. 68, Table 2) showed normal ALT values. As shown in Table 3, elevated ALT levels were highly correlated with the HEV-RNA, HEV-Ag, HEV-IgM but not with the HEV-IgG and HEV-Total Ab. Yet, 64 (27.2%) of the samples had elevated ALT with negative HEV RNA and IgM results.

**Table 2:** RT-PCR confirmed HEV Patients’ demographic data, ALT, RT-PCR and serology results

Sample no	ALT	Nationality	Wantai HEV-IgG	Wantai HEV-IgM	MP HEV-Total Ab	CT-value**	Wantai HEV-Ag
34	2766	Indian	+	+	+	+	+
43	1314	Indian	+	+	+	(++) 23	+
44	2346	Indian	+	+	+	(+) 32	+
45	3569	Indian	−	+	+	(++) 22	+
46	40	Indian	+	+	+	(++) 29	−
49	ND	Egyptian	+	−	+	(+++)10	−
50	919	Bangladeshi	+	+	+	(+) 31	−
52	ND	Pakistanis	+	+	+	(+)36	+
53	ND	Bangladeshi	+	+	+	(+) 30	+
54	1321	Bangladeshi	+	+	+	(+) 31	−
55	107	Qatari	−	−	−	(+++)19	−
63	146	Egyptian	−	−	+	(+) 39	−
66	42	Nepalese	−	−	−	(+) 31	−
67	379	Indian	−	−	−	(+) 36	−
68	15	Qatari	−	−	−	(+) 39	−
72	947	Indian	+	ND*	+	(+) 30	+
74	396	Indian	+	+	+	(+) 32	−
75	1122	Indian	+	+	+	(+) 34	+
85	ND	Indian	ND	ND	ND	(++) 28	ND
86	91	Pakistanis	ND	ND	ND	(+) 38	ND
87	99	Pakistanis	−	+	+	(++) 20	−
88	103	Sudanese	+	+	+	(+) 30	−
89	ND	Nepalese	+	+	+	(+) 30	−
90	95	Siri Lankan	−	+	−	(+) 32	−

ALT reference range: 0 U/L–41 U/L

*ND Not done because of no sufficient sample

**(+++) or highly positive: CT value < 20; (++) or moderately positive: CT value from 20 to 30; (+) weakly positive: CT value between 30 and 40; (−) or negative CT value less than 45 considered negative

**Table 3:** Summary of the positive results obtained by each assay to detect the presence of HEV among non-A-C hepatitis patients in Qatar (n = 259)

HEV marker	No. positive (%)	Total no. of ALT tested specimen	Sample no. with normal ALT (%)	Sample no. with an elevated ALT (%)
Wantai-IgM	20 (8.65)	17	2/17 (11.8)	15/17 (88.2)
Wantai HEV-IgG	74 (32.1)	69	17/69 (24.6)	51/69 (73.9)
Wantai HEV-Ag	10 (5.56)	8	0 (0)	8/8 (100)
MP HEV-Total Ab	53 (34.2)	49	13 (26.5)	36/49 (37.5)
RT-PCR HEV-RNA	24 (23.1)	19	1 (5.3)	18/19 (94.7)

ALT test was not done in all samples due to insufficient volume

### HEV-RNA and serological markers association with age, gender, and race

In regard to age, as expected, HEV-IgG seropositivity increased significantly with age (Table 1); seroprevalence peaked among 55+ year-olds (50%), compared with 28.9% among those < 24 years of age (*p* < 0.024). A similar trend was observed with HEV-Total Ab; the highest rate of HEV-Total Ab seroprevalence was observed in the eldest group (55+, 62.5%). Incontrast, HEV-IgM serevoprevelence peaked (21.1%) in the youngest age group (< 24 years of age). Overall, HEV-IgG, HEV-IgM, HEV-Ag, and HEV-Total Ab seropositivity were significantly associated with age in non-A-C hepatitis patients in Qatar (*p* < 0.05) as shown in Table 1.

**Fig. 1:**
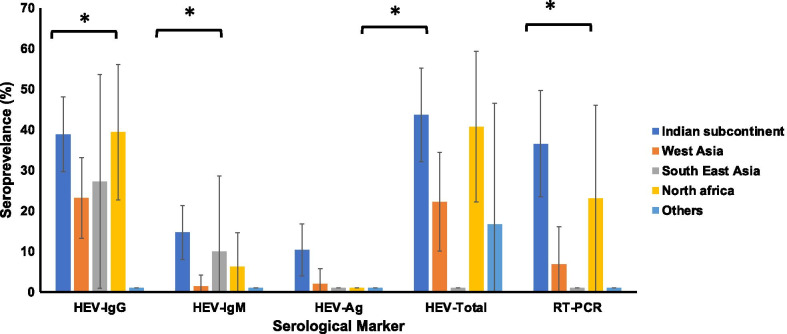
Representative figure of the seroprevalence (with 95% confidence intervals) of HEV-IgG, HEV-IgM, HEV-Ag, HEV-Total Ab and RT-PCR in non-A-C hepatitis patients in Qatar. Indian Subcontinent includes Bangladesh, India, Nepal, Pakistan, and Sri Lanka. Western Asia includes Syria, Lebanon, Jordan, Palestine, Oman, Iran, Bahrain, Saudi Arabia, Yemen, and Qatar. Southeast Asia includes Philippines, and Myanmar. North Africa includes Sudan, Algeria, Tunisia, Sudan, and Egypt. Other includes Eretria, Spain, America, Europe, Uganda, Somalia, Cuba, and Mexico N = 259. *p < 0.05

With regard to gender and race, HEV-IgG seropositivity was significantly associated with gender (*p* = 0.01); 38.3% of males and 22.4% of females were positive for HEV-IgG antibodies. In addition, HEV-IgG seropositivity was statistically associated (*p* = 0.03) with race, where 38.8% and 39.4% of the patients were coming from the Indian subcontinent group and North African, respectively. Similarly, the seroprevalence of HEV-IgM and HEV-Total Ab were significantly higher among males (*p* = 0.0001 and *p* = 0.025, respectively) compared to females. In addition, a significant association was observed between the seropositivity of HEV-IgM with race, where the highest seroprevalence was observed in patients coming from the Indian subcontinent area. Further, a significant association was found between samples testing positive for HEV-RNA and having been born in the Indian subcontinent region (36.5%, *p* = 0.03) (Fig. 1, Table1).

### Sensitivity, specificity, and concordance of HEV serological assays compared to RT-PCR

We assessed the performance of HEV-Ag, HEV-IgG, HEV-IgM, and HEV-Total Ab test to be used as a complementary test to RT-PCR to improve its diagnostic sensitivity in clinical settings. The sensitivity, specificity, predictive values, and concordance of these assays were evaluated in comparison with the RT-PCR reference assay. MP HEV-Total Ab assay showed the highest sensitivity of 77.3%, followed by Wantai HEV-IgM (71.4%), Wantai HEV-IgG (63.6%), and Wantai HEV-Ag (36.3%). Although Wantai HEV-Ag showed the lowest sensitivity (36.3%), yet, it had the highest specificity (100%). The overall percent agreement was 91.0% for Wantai HEV-IgM, followed by 81.1% for Wantai HEV-Ag, 73.2% for MP HEV-Total Ab, and 70.3% for Wantai HEV-IgG. Importantly, the agreement with RT-PCR results based on Cohen’s kappa was as follows: Wantai IgM ELISA (0.71) > MP HEV-Total Ab and Wantai HEV-Ag (0.44) > Wantai HEV-IgG (0.29). Therefore, based on the overall Cohen kappa and overall agreement with RT-PCR, MP HEV-Total and Wantai HEV-IgG showed the poorest performance and agreement with RT-PCR.

## Discussion

Acute HEV infection can be diagnosed by the detection of anti-HEV antibodies (IgM, IgG or both) by enzyme immunoassays. However, till now, there are plethora of issues regarding the performance of HEV serological assays that require urgent attention, such as, the specificity of certain assays is not optimal, cross reactivity with other viruses (EBV and CMV), and anti-HEV IgM on its own is not a sufficiently robust marker for diagnosis [34]. Therefore, serology assays alone are unreliable in the diagnosis of acute viral hepatitis. Thus, the European Association of the Study of the Liver (EASL) recommends using a combination of serology and nucleic acid test (NAT) testing to diagnose HEV infection in immunocompetent patients, while it recommends NAT testing to diagnose chronic HEV infection in immunocompromised patients [35]. In the present paper, we conducted a study on sporadic cases of acute non-A-C hepatitis in Qatar by comparing the performance of four dominant, commercially available Wantai HEV-IgM, Wantai HEV-IgG, Wantai HEV-Ag assays, and MP HEV-Total Ab test to RT-PCR to evaluate the current status of their performance.

Acute HEV infections were identified in 23.1% of the non-A-C hepatitis patients in our study. However, this prevalence of HEV-RNA could be underestimated. That is, the HEV-RNA can be detected in serum only during the viremia stage and last for a very short period during the early convalescence stage [3, 9, 26]. For instance, three samples in our study showed positive HEV-IgM, HEV-IgG, HEV-Total Ab and high ALT value, but negative RT-PCR results. These 3 patients could be in an early convalescence stage, where RNA disappeared or could be due to the low level of HEV-RNA in their serum [36]. As the HEV-RNA detected for a longer period in the stool than the blood during the acute stage [37], any future study should include stool samples from these patients for better estimation of the HEV prevalence.

In the 24 RNA positive specimens that were tested by all assays, HEV antibodies were identified in 66.7% (16/24) of the patients, at least, by two serological assays, as shown in Table 2. Thus, in agreement with the dynamic of acute infection, 66.6% of patients were in the early post-seroconversion stage (all three markers positive) [38]. Only three patients (No. 55, 66, and 67) were in the window period of the acute phase where antibodies were not yet detectable, and viremia and increased ALT values were the only markers of infection. Another reason could be that these patients might not have elicited enough antibody response as the samples were collected too early to be positive during the acute phase. These results suggest that ALT could also be used as a good acute HEV marker as well. On the other hand, one sample (No. 68) showed positive RT-PCR test, negative serology testing and normal ALT level (15 IU/L). This sample most likely belongs to an asymptomatic patient.

Zhang et al. has indicated that HEV-Ag in macaques became detectable in the serum at almost the same time as HEV-RNA in feces [28]. They and others suggested that HEV-Ag detection should be a valuable tool for the diagnosis of acute HEV, particularly in the window period before seroconversion to anti-HEV [28, 33]. To our knowledge, Wantai Ag-ELISA is the only commercial assay that is currently present in the market for the diagnosis of HEV Ag. Our study is one of the very few studies that evaluated the performance of HEV Ag for the diagnosis of acute HEV [11, 39, 40]. However, our results showed that the sensitivity Wantai Ag-ELISA was the lowest (36.4%) compared to the other serological assays, as shown in Table 4, suggesting that the Wantai HEV-Ag might not be very useful to be used as a single screening assay. In addition, the Wantai HEV-IgM and MP HEV-Total Ab conventional assays showed better sensitivity of 71.4% and 77.3%, respectively, suggesting that Wantai HEV-IgM and MP HEV-Total Ab assays for diagnosis of acute HEV are superior to HEV-Ag assay. Our study confirms the findings of a recent study conducted by Vollmer et al., where they evaluated the performance of Wantai HEV-Ag and HEV-IgM assays for the detection of HEV-Ag and HEV-IgM in positive blood donors in comparison to the RT-PCR assay [11, 40]. In Vollmer’s study, Wantai HEV-Ag was able to detect HEV-Ag only in 40% (4/10) of the positive HEV-RNA donors. In addition, HEV-IgM was detected in 70% (7/10) of the same donors, which are in agreement with our results. In contrast, two other studies had demonstrated that Wantai HEV-Ag assay could be used as an alternative early detection marker for the diagnosis of acute HEV [39–41] and HEV-Ag demonstrated a good concordance with HEV-RNA, while the presence of HEV-IgM did not demonstrate any concordance with HEV-RNA [39]. However, our findings, along with Vollmer et al. results, showed a significant diagnostic gap between the presence of HEV-RNA and HEV-Ag (kappa 0.44) by ELISA and to a lesser degree, with HEV-IgM by ELISA tests (kappa 0.71).

**Table 4:** Assays performance according to the RT-PCR assay

Kit	Overall agreement % (95% CI)	Sensitivity % (95% CI)	Specificity % (95% CI)	PPV % (95% CI)	NPV % (95% CI)	Cohen’s Kappa value (CI)
Wantai HEV-Ag	81.1 (70.7–88.4)	36.7 (16.3–56.5)	100.0 (100–100)	100.00 (100–100)	78.7 (68.9–88.7)	0.44 (0.22–0.66)
Wantai HEV-IgM	91.0 (83.8–95.2)	71.4 (52.1–90.8)	96.20 (91.9–100)	83.3 (66.1–100.5)	92.6 (87.0–98.3)	0.71 (0.53–0.88)
Wantai HEV-IgG	70.3 (60.8–78.3)	63.64(43.5–83.7)	72.15 (62.3–82.0)	38.8 (22.9–54.8)	87.6 (79.7–95.6)	0.29 (0.10–0.48)
MP HEV-Total Ab	73.2 (61.9–82.1)	77.27 (59.8–94.8)	71.43 (58.8–84.1)	54.8 (37.3–72.3)	87.5 (77.2–97.7)	0.44 (0.23–0.64)

The overall percent agreement is the percentage of total subjects where the new test and the non-reference standard agree

*PPV* positive predictive value, *NPV* negative predictive value

The seroprevalence of MP HEV-Total Ab (IgG, IgM and IgA) was the highest among non-A-C hepatitis patients (34.2%) followed by Wantai HEV-IgG (32.1%) and Wantai HEV-IgM (8.65%) as shown in Table 1. Our results are almost similar to the recent study [22], where they reported high seroprevalence for HEV-IgG (18.0%) among blood donors in Qatar. However, as expected, HEV-IgM was much higher in acute non-A-C hepatitis patients (8.65%) compared to blood donors (only 0.20%) [22]. In this study, we included participants from 32 different countries. Most of the migrants resides in Qatar for a minimum of 2 years. We showed here that highest HEV-IgG antibody seroprevalence belonged to participants from Bangladesh, Egypt, Nepal, India, Pakistani, Sudan, Philippine, and SriLanka with seroprevalences of 50, 47.6, 47.5%, 37.5, 36.4, 30.0, 30.0, and 20%, respectively. Meanwhile, Qatar comes at a much lower seroprevalence of 17% as shown in Additional file 1: Table S2. These results were consistent with our previous study, in which, we included 5854 blood donor participants from more than 100 different countries. The highest HEV antibody seroprevalence belonged to blood donor participants from Sudan, Pakistan, Egypt, Yemen, Syria, and India with seroprevalences of 51.5, 40.9, 38.8%, 18.8, 15.8, and 15.1 respectively. Similar to our findings, Qatar comes at a much lower seroprevalence of 11.5%, even though Qataris were the largest community of blood donation [42]. The prevalence of HEV antibody–positive cases among non-Qataris is nearly double that of Qataris (22.9% vs. 11.5%, respectively), with a significant statistical association (*p* < 0.001)[42].

Even though the Wantai HEV-IgG resulted in a significantly higher seroprevalence (Table 3), it showed the weakest performance compared to the rest of the assays (sensitivity = 63.6%, specificity = 72.1%, and kappa = 0.29). The reason behind this might be because all the samples were collected in the acute phase of the infection, where HEV-IgG immunoglobulins are yet below the detectable limits. In other words, HEV IgG appears shortly after the IgM response, which appears one week to two months after the onset of illness. Similar to our results, in a series of 44 children with acute HEV (confirmed with HEV viremia in serum and stool by cell culture and RT-PCR), only 35 percent of patients tested positive for HEV-IgM in serum and only 3 percent were positive for HEV-IgG [43]. In another study, HEV-RNA was detected in 23% of patients, followed by the detection of specific HEV-IgM in 17% and HEV-IgG in 13% of patients. This might explain the high discordance between assays of HEV-IgG antibody (depending on the time of sample collection) as compared with assays for HEV-IgM antibody [44]. Another reason, IgG positivity could be due to a previous infection, especially those that are HEV-RNA and HEV-IgM negative samples.

HEV usually causes acute hepatitis; however, it also can develop chronic infection [4]. HEV has 4 main well established and 3 recently discovered genotypes, which all have dictint epidemiological distinct pattern based on socioeconomic factors and ecology [45, 46]. All HEV genotypes can cause human infections. HEV‐1 and HEV-2 solely infect humans and lack any zoonotic origin. HEV-1 accounts for epidemics in parts of Asia [9], while HEV‐2 is more prevalent in Africa, Mexico, and other developing countries. In countries with poor resources and low sanitation such as India, Bangladesh, Sri Lanka, Egypt, Mexico, and China, HEV‐1 and HEV‐2 infections manifest as large‐scale water-borne epidemics (fecal–oral transmission) and have spread through person‐to‐ person contact [4, 47]. On the other hand, HEV-3 and HEV-4 have zoonotic origins and primarily infect pigs, boars and deer. HEV‐3 and HEV‐4 are transmitted zoonotically in developed countries such as Japan, USA, and several European countries with sporadic cases [4, 47]. HEV‐3 is prevalent worldwide, while HEV‐4 is mainly prevalent in Asia. Both HEV‐3 and HEV‐4 are transmitted through contact with infected animals and by consumption of contaminated raw or undercooked beef, pork, or shellfish [4, 47]. Finally, HEV‐5 and HEV‐6 are novel genotypes identified in wild boar in Japan [48, 49], while, HEV‐7, a newly discovered HEV genotype, primarily infects dromedary camels (Arabian 1‐humped camels) and was identified in the UAE very recently [45]. To this end, as most of HEV-RNA-positive cases in our study belong to non-Qatari residents, particularly, from the Indian subcontinent (India, Pakistan and Bangladesh), we expect that most prevalent genotype would be HEV-1, followed by HEV-3. As shown Table 2, 19 samples out of the 24 HEV positive samples (79%) were from immigrant workers coming from South Asia (Bangladesh, India, Nepal, Pakistan and Sri Lanka). Recently a new genotype 7 was isolated in UAE (close to Qatar) from both camels and humans, yet it is now known if this genotype would also be found in Qatar [50, 51]. Indeed, several studies from Europe and Italy, targeted HEV prevalence in migrant population, have shown that HEV-1 is the most imported and prevalent genotype among Asian migrants such Indian, Pakistani Bengali and Nepali migrants[52–55].

Overall, our data suggest that HEV-IgM positivity represents the main biological marker of HEV acute infection in the clinical setting of developed countries as it showed the best overall performance and best concordance with RT-PCR with a kappa value of 0.71, which denotes substantial agreement. Although Wantai HEV-Ag is the only commercial assay that is currently present in the market for the diagnosis of acute HEV-Ag, the employment of this assay with such a low sensitivity (36.36%) could erroneously fail to confirm HEV-IgM results and could cause drastic underestimation of acute HEV infection cases [56].

## Conclusion

HEV leads to outbreaks in developing countries and causes significant morbidity and mortality in immunocompromised and pregnant females. Our knowledge of HEV infection has increased dramatically over the past 20 years. HEV laboratory findings and clinical symptoms related to HEV infection are vague and common among other hepatitis infections. In addition, the use of HEV-antibodies diagnostic tests of low specificity and sensitivity has made HEV diagnosis difficult and challenging. We recommend performing routine RT-PCR HEV in all cases of acute non-A-C hepatitis. However, a combination of serology and RT-PCR is the recommended testing strategy for suspected patients, when applicable. Thus early detection accurate diagnosis of HEV infection. Although we observed significant inconsistencies between different serological assay kits and HEV RNA assay, caution is warranted while interpreting the results of both serological and RT-PCR in HEV diagnoses. We believe that the knowledge of the analytical sensitivity towards all the HEV genotypes gains fundamental importance to assess the reliability of the test in HEV acute infection diagnosis, especially during an outbreak and other emergencies in countries with limited resources.

## Supplementary Information

**Additional file 1: Table S1**. Overview of specifications for the different HEV immunoassays. **Table S2**. Difference of anti HEV seroprevalence between different nationalities. **Table S3**. Demographic characteristics of the 259 included patients.

## Data Availability

To ensure patient confidentiality, raw data supporting our findings will be available upon request from the corresponding author: Dr Gheyath K. Nasrallah.

## Abbreviations

RT-PCR: Reverse transcription polymerase chain reaction
ELISA: Enzyme immune assay
ALT: Alanine aminotransferase
CT: Cycle threshold
PPV: Positive predictive values
NPV: Negative predictive values
CI: Confidence interval
