# Supplementary material for: The prevalence of HEV among non-A-C hepatitis in Qatar and efficiency of serological markers for the diagnosis of hepatitis E
Source: BMC Gastroenterol. 2021 Jun 15;21:266. doi: 10.1186/s12876-021-01841-2 (PMC8207580; doi:10.1186/s12876-021-01841-2)
Supplement: Supplementary file 1 — Additional file 1: Table S1. Overview of specifications for the different HEV immunoassays. Table S2. Difference of anti HEV seroprevalence between different nationalities. Table S3. Demographic characteristics of the 259 included patients. [file 12876_2021_1841_MOESM1_ESM.docx]

Table S1 Overview of specifications for the different HEV immunoassays.

| Anti-HEV ELISA | Assay Type | Antigen/Origin | Analysis and Serostatus Interpretation |
| --- | --- | --- | --- |
| Wantai HEV Ag ELISA | Qualitative, direct | HEV antigen | - negative: C.O. < 1  - borderline: C.O. 0.9–1.1  - positive: C.O. ≥ 1 |
| Wantai HEV IgM ELISA | Qualitative, μ-chain capture | Recombinant antigen ORF-2 C-terminal, genotype 4 | - negative: C.O. < 0.9  - borderline: C.O. 0.9–1.1  - positive: C.O. ≥ 1.1 |
| Wantai HEV IgG ELISA | Qualitative, indirect | Recombinant antigen ORF-2 C-terminal, genotype 4 | - negative: C.O. < 0.9  - borderline: C.O. < 0.9–1.1  - positive: C.O. ≥ 1.1 |
| HEV ELISA 4.0 (MP Biomedicals) - IgG and IgM | Qualitative, direct | 1 recombinant antigen, ORF-2 C-terminal  (Chinese strain), genotype 1 | - negative: C.O. < 0.4 + NRC  - positive: C.O. ≥ 0.4 + NRC |

Table S2 difference of anti HEV seroprevalence between different nationalities.

|  | IgG seroprevelance (%) | IgM seroprevelance  (%) |
| --- | --- | --- |
| Qatari | 17.0 | 2.13 |
| Indian | 37.5 | 16.7 |
| Bangladeshi | 50.0 | 25.0 |
| Egyptian | 47.6 | 5.00 |
| Nepalease | 43.5 | 4.17 |
| Pakistani | 36.4 | 18.2 |
| Sudanese | 30.0 | 10.0 |
| Philipino | 30.0 | 11.1 |
| Srilankan | 20.0 | 10.0 |
| others | 23.5 | 0 |

Table S3 Demographic characteristics of the 259 included patients

| Indian Subcontinent | |
| --- | --- |
| Sample No. | **Nationality** |
| 1 | Nepalease |
| 2 | pakistani |
| 4 | Indian |
| 5 | Nepalease |
| 9 | Bangladeshi |
| 10 | Nepalease |
| 11 | Nepalease |
| 12 | Nepalease |
| 13 | Indian |
| 16 | Naplaese |
| 17 | Pakistani |
| 25 | Indian |
| 26 | Banghadishi |
| 28 | Banghadishi |
| 34 | Indian |
| 39 | Naplaese |
| 42 | Indian |
| 43 | Indian |
| 44 | Indian |
| 45 | Indian |
| 46 | Indian |
| 47 | Banghadishi |
| 50 | Banghadishi |
| 52 | Pakistani |
| 53 | Banghadishi |
| 54 | Banghadishi |
| 59 | Indian |
| 60 | Naplaese |
| 62 | Indian |
| 65 | Indian |
| 66 | Naplaese |
| 67 | Indian |
| 69 | Naplaese |
| 70 | Indian |
| 72 | Indian |
| 74 | Indian |
| 75 | Indian |
| 76 | Indian |
| 79 | Indian |
| 80 | Naplaese |
| 81 | Indian |
| 85 | Indian |
| 86 | Pakistani |
| 87 | Pakistani |
| 89 | Naplaese |
| 90 | Srilankan |
| 92 | Nepalease |
| 98 | Srilankan |
| 101 | Nepalease |
| 103 | Srilankan |
| 105 | Indian |
| 112 | Indian |
| 113 | Indian |
| 114 | Bangladeshi |
| 115 | Bangladeshi |
| 117 | Srilankan |
| 118 | Indian |
| 119 | pakistani |
| 125 | Indian |
| 127 | Indian |
| 130 | Indian |
| 132 | pakistani |
| 142 | pakistani |
| 146 | Indian |
| 147 | Sirilankan |
| 149 | pakistani |
| 155 | Indian |
| 156 | Nepalease |
| 157 | Indian |
| 158 | Bangladeshi |
| 159 | Indian |
| 161 | Bangladeshi |
| 162 | Indian |
| 165 | Nepalease |
| 166 | Indian |
| 167 | Sirilankan |
| 172 | Nepalese |
| 173 | Srilankan |
| 176 | Indian |
| 177 | Nepalese |
| 178 | Bangladeshi |
| 180 | Indian |
| 187 | Srilankan |
| 188 | Indian |
| 189 | Indian |
| 190 | Indian |
| 191 | Indian |
| 194 | Pakistani |
| 197 | Pakistani |
| 198 | Bangladeshi |
| 200 | Indian |
| 201 | Bangladeshi |
| 202 | Nepalese |
| 206 | Nepalese |
| 208 | Indian |
| 209 | Nepalese |
| 211 | Srilankan |
| 212 | Indian |
| 217 | Indian |
| 220 | Nepalese |
| 224 | Nepalese |
| 227 | Pakistani |
| 228 | Bangladishi |
| 229 | Indian |
| 230 | Bangladishi |
| 232 | Indian |
| 234 | Indian |
| 235 | Pakistani |
| 236 | Srilankan |
| 238 | Indian |
| 241 | Indian |
| 243 | Indian |
| 244 | Srilankan |
| 245 | Indian |
| 246 | Nepalese |
| 247 | Bangladishi |
| 250 | Neplese |
| 253 | Bangladishi |
| 255 | Indian |
| 258 | Indian |
| 259 | Indian |
| West Asia | |
| 3 | Qatari |
| 6 | Qatari |
| 7 | Qatari |
| 15 | Lebanese |
| 18 | Qatari |
| 19 | Syrian |
| 22 | Syrian |
| 24 | Qatari |
| 27 | Qatari |
| 29 | Qatari |
| 33 | Qatari |
| 36 | Qatari |
| 37 | Iranian |
| 55 | Qatari |
| 58 | Qatari |
| 61 | Qatari |
| 64 | Qatari |
| 68 | Qatari |
| 71 | Qatari |
| 73 | Qatari |
| 77 | BAH |
| 78 | Qatari |
| 84 | Qatari |
| 91 | Saudi |
| 94 | Qatari |
| 95 | Qatari |
| 96 | Saudi |
| 97 | Qatari |
| 102 | Palestinian |
| 104 | Palestinian |
| 107 | Qatari |
| 108 | Jordinian |
| 110 | Qatari |
| 116 | Qatari |
| 120 | Qatari |
| 121 | Syrian |
| 122 | Qatari |
| 124 | Palestinian |
| 126 | Qatari |
| 128 | Qatari |
| 133 | Qatari |
| 134 | Qatari |
| 135 | Qatari |
| 136 | Qatari |
| 139 | Iranian |
| 141 | Qatari |
| 143 | Qatari |
| 145 | Qatari |
| 150 | Qatari |
| 151 | Yemeni |
| 153 | Qatari |
| 154 | Syrian |
| 163 | Omani |
| 168 | Qatari |
| 174 | Qatari |
| 175 | Qatari |
| 179 | Qatari |
| 184 | Qatari |
| 186 | Jordinian |
| 195 | Qatari |
| 196 | Qatari |
| 203 | Iranian |
| 204 | Jordinian |
| 213 | Qatari |
| 215 | Yamani |
| 216 | Qatari |
| 219 | Kuwaiti |
| 221 | Qatari |
| 222 | Qatari |
| 226 | Qatari |
| 231 | Iranian |
| 237 | Saudi |
| 242 | Qatari |
| 248 | Iranian |
| 251 | Qatari |
| 252 | Qatari |
| 254 | Yemni |
| 256 | Jordanian |
| 257 | Qatari |
| Southeast Asia | |
| 21 | Philipino |
| 23 | philipino |
| 40 | Philipino |
| 56 | PHI |
| 100 | Mynmar |
| 138 | Philipino |
| 170 | Philipino |
| 181 | Philipino |
| 185 | Philipino |
| 210 | Philipino |
| 218 | Philipino |
| 249 | Philipino |
| North Africa | |
| 14 | Egyptian |
| 20 | Sudanese |
| 30 | Egyptian |
| 31 | Sudanese |
| 41 | Egyptian |
| 48 | Sudanese |
| 49 | Egyptian |
| 51 | Egyptian |
| 57 | Sudanese |
| 63 | Egyptian |
| 82 | Egyptian |
| 83 | Egyptian |
| 88 | Sudanese |
| 93 | Sudanese |
| 99 | Egyptian |
| 106 | Tunisian |
| 109 | Egyptian |
| 111 | Egyptian |
| 123 | Egyptian |
| 129 | Sudanese |
| 131 | Egyptian |
| 137 | Sudanese |
| 140 | Egyptian |
| 148 | Sudanese |
| 152 | Egyptian |
| 160 | Egyptian |
| 164 | Algerian |
| 169 | Egyptian |
| 171 | Egyptian |
| 192 | Egyptian |
| 193 | Egyptian |
| 199 | Egyptian |
| 205 | Sudanese |
| 225 | Eygyptian |
| 233 | Eygptian |
| 240 | Eygyptian |
| Others | |
| 8 | Eritrean |
| 32 | Spanish |
| 35 | Ghanaian |
| 38 | Italian |
| 144 | Somalian |
| 182 | Maxican |
| 183 | Cuban |
| 207 | Cuban |
| 214 | Cameroonian |
| 223 | Ugandan |
| 239 | American |
